# Vitelline Membrane Protein 26 Mutagenesis, Using CRISPR/Cas9, Results in Egg Collapse in *Plutella xylostella*

**DOI:** 10.3390/ijms23179538

**Published:** 2022-08-23

**Authors:** Yi-Long Zhai, Shi-Jie Dong, Ming-Min Zou, Yu-Dong Qin, Li-Li Liu, Min-Hui Cao, Meng-Qi Huang, Liette Vasseur, Min-Sheng You, Lu Peng

**Affiliations:** 1 State Key Laboratory of Ecological Pest Control for Fujian-Taiwan Crops, Institute of Applied Ecology, Fujian Agriculture and Forestry University, Fuzhou 350002, China; 2 Ministerial and Provincial Joint Innovation Centre for Safety Production of Cross-Strait Crops, Fujian Agriculture and Forestry University, Fuzhou 350002, China; 3 Key Laboratory of Integrated Pest Management for Fujian-Taiwan Crops, Ministry of Agriculture, Fuzhou 350002, China; 4 Fujian Provincial Key Laboratory of Insect Ecology, Fujian Agriculture and Forestry University, Fuzhou 350002, China; 5 Department of Biological Sciences, Brock University, St. Catharines, ON L2S 3A1, Canada

**Keywords:** diamondback moth, egg formation, mutation, gene expression, embryonic development

## Abstract

Vitelline membrane proteins (VMPs) are the main proteins that form the inner shell (vitelline membrane layer) of insect eggs and are an integral part of egg formation and embryo development. Here, we characterized the molecular structure and expression patterns of the *VMP26* gene and analyzed its reproductive functions in diamondback moth, *Plutella xylostella* (L.), a worldwide migratory pest of cruciferous plants. The *PxVMP26* gene was shown to be a single exon gene that contained an open reading frame of 852 base pairs (bp) encoding 283 amino acids. Both qPCR and western blot analyses showed that *Px*VMP26 was specifically expressed in female adults and was significantly highly expressed in the ovary. Further anatomical analysis indicated that the expression level of *PxVMP26* in the ovarian tube with an incomplete yolk was significantly higher than that in the ovarian tube with a complete yolk. CRISPR/Cas9-induced *PxVMP26* knockout successfully created two homozygous strains with 8- and 46-bp frameshift mutations. The expression deficiency of the *Px*VMP26 protein was detected in the mutant strains using immunofluorescence and western blot. No significant difference was found in the number of eggs laid within three days between wild and mutant individuals, but there was a lower egg hatchability. The loss of the *PxVMP26* gene changed the mean egg size, damaged the structure of the vitelline membrane, and increased the proportion of abnormal eggs due to water loss, resulting in egg collapse. This first analysis of the roles of the *VMP* gene in the oocyte formation and embryonic development of *P. xylostella*, using CRISPR/Cas9 technology, provides a basis for screening new genetic control targets of *P. xylostella*.

## 1. Introduction

The formation of eggshell structure is the last step in insect oogenesis. This process is essential to protect the embryo from mechanical damage and microbial infection, prevent water loss, allow gas exchange, and participate in embryonic polarity formation [1]. Although eggs are diverse in structure and function [2,3], the eggshell layers are common among insect groups and conserved in their arrangement pattern [4,5]. There are five clearly defined layers from the oocyte to the outer surface, in order of the vitelline membrane, inner chorion layer, endochorion, exochorion, and chorion [6,7]. 

The vitelline membrane is the first layer secreted by the follicular cells [4,8]. It is located on the oocyte surface and interacts with the oocyte membrane [3,4]. The vitelline membrane is essential in insect oogenesis as it does not only play the same protective function as the outer eggshell, but also performs a decisive role in water retention and gas exchange [4,8,9]. The vitelline membrane contains a variety of proteins, among which vitelline membrane proteins (VMPs) are the main components. They are synthesized and secreted by follicular epithelium cells in the insect’s ovary [10,11]. Five *VMP* genes have been characterized in *Drosophila melanogaster* through high-throughput analysis [12]. They are mainly small molecular proteins rich in proline and contain a conserved domain of hydrophobic C terminus (VM domain) [13]. Three novel *VMPs* are reported in *Aedes aegypti* by the analysis of transcriptome and proteome, including *VMP15a-1/2/3* [14]. Two *VMPs* are found in *Anopheles gambiae* [15]. In *Bombyx mori*, six putative *VMP* genes (*VMP15a-1/2/3*, *VMP30*, *VMP90*, and *EP80*) have been identified, and three *VMP* genes in *Anastrepha obliqua* [16], *Bactrocera dorsalis* [17,18], and *Zeugodacus cucurbitae* [19]. 

Studies of the functions of insect *VMPs* have mainly targeted model insects [20,21,22]. For example, *VMP* inhibition results in abnormal dorsal–ventral polarity, deficient female fertility, and disrupted embryonic development in *D. melanogaster* [20,22,23,24]. *VMPs*, such as *VMP23*, *VMP30*, *EP80*, and *VMP90*, are reported to play indispensable roles in the follicular integrity of *B. mori* [25,26,27,28,29]. In *B. mori*, the loss of *BmEP80* causes the collapse of eggs, resulting in the death of the embryo due to water loss [26,28]. *BmVMP30* deficiency results in the binding of the follicles to the outer basal membrane, which affects the integrity of the oocyte [29]. Inhibition of *BmVMP90* expression induces an abnormal follicular phenotype with detached follicular epithelia, suggesting the significance of *BmVMP90* in the integrity of developing follicles [27].

The diamondback moth, *Plutella xylostella* (L.) (Lepidoptera, Plutellidae), is a major pest of cruciferous plants that causes huge economic losses to crop production. While not considered a model insect, in recent years research has intensified as its high fecundity enables it to invade any areas where cruciferous plants grow, making it one of the most widespread lepidopteran pests in the world [30]. Understanding its oogenesis may help find ways to reduce its reproductive ability. Three *VMP* genes have been predicted from the transcriptome and genome data of *P. xylostella* [31,32]. To date, however, the functions of these *VMP* genes have yet to be elucidated. Considering that an effective RNAi is difficult to obtain in most lepidopteran insects [33], the technology of CRISPR/Cas9 (clustered regularly interspaced short palindromic repeat/CRISPR-associated nuclease 9) can be effective to explore the gene functions in vivo [34,35]. Thus far, CRISPR/Cas9-mediated validations of gene functions have been achieved in *D. melanogaster*, *Anopheles gambiae*, *B. mori*, *P. xylostella*, and other agricultural pests [36,37,38,39,40].

In this study, the molecular characteristics of *P. xylostella VMP26* were identified, and its temporal and spatial expression profiles were analyzed. CRISPR/Cas9-mediated knockout of *PxVMP26* was performed to investigate its functions in the oogenesis and embryonic development of *P. xylostella*. Our results confirmed the crucial roles of *VMP26* in the eggshell formation of *P. xylostella*, suggesting that this gene could serve as a novel genetic-based molecular target for pest control.

## 2. Results

### 2.1. Identification and Analysis of PxVMP26

**Figure 1:**
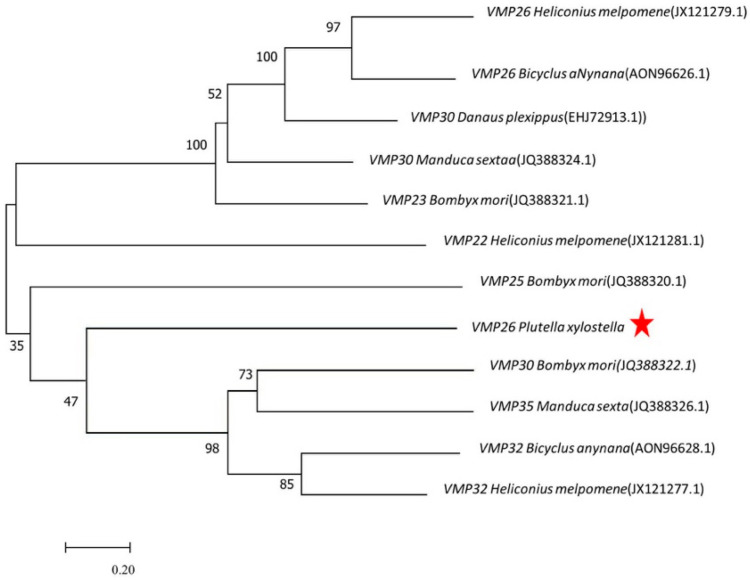
Phylogenetic relationship of *Px*VMP26 and the small molecular VMPs of other Lepidoptera species. The phylogenetic tree was constructed by MEGA 7 using the neighbor joining method with 1000 bootstrap replicates. The VMP26 in *P. xylostella* is indicated by the red star.

*PxVMP26* consisted of a single exon that contained an open reading frame (ORF) of 852 base pairs (bp) encoding for 283 amino acids (Figure S2). The theoretical molecular mass was estimated at 26 kDa. *Px*VMP26 did not contain a VM conservative domain, but it was rich in leucine, serine, alanine, and proline. The phylogenetic analysis showed that the small molecular VMPs of Lepidoptera evolved into two large branches, and further differentiated into four subclades, among which *Px*VMP26 was clustered into a single subclade. *Px*VMP26 was closely related to *B. mori* VMP30 (Figure 1).

### 2.2. Expression Profile of PxVMP26

**Figure 2:**
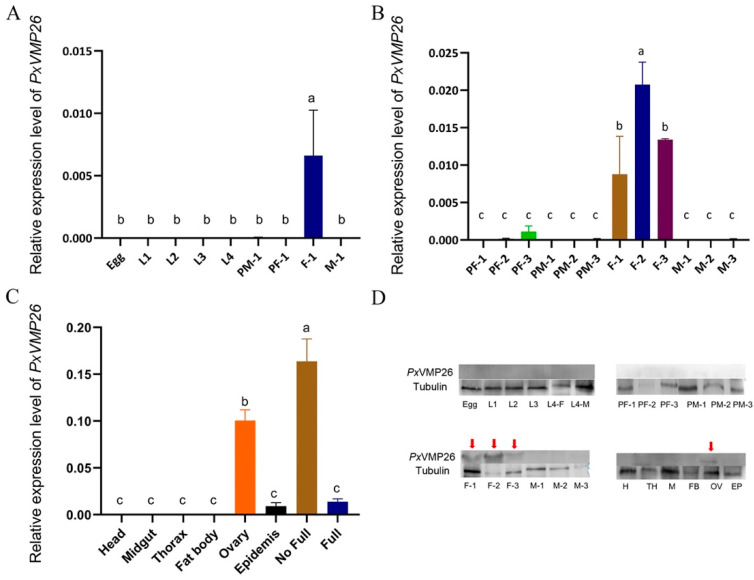
The developmental and tissue-specific expression profiles of the *PxVMP26* in *P. xylostella*. (**A**–**C**) The expression profiles of *PxVMP26* transcripts were analyzed by qRT-PCR; the mRNA level was normalized to ribosomal protein genes L32 (RPL32), L8 (RPL8), and elongation factor 1 alpha (EF-1α). Data shown as mean ± SE representing three biological replicates. Different letters on the bars indicate significant differences (*p* < 0.05) using LSD’s multiple range test. (**D**) The expression profiles of *Px*VMP26 protein were analyzed by western blot. The proteins from different stage (30 ug) or tissue (20 ug) was separated using 15% SDS-PAGE, and tubulin was used as the internal reference. L1–4, 1–4 instar larvae; PF/PM-1/2/3, male and female pupae 1/2/3 d; F/M-1/2/3, male and female adults 1/2/3 d; no full, ovarian tube with incomplete yolk deposition; full, ovarian tube with complete yolk deposition. The VMP26 protein is indicated by the red arrows.

The developmental expression profile showed that *PxVMP26* was specifically expressed in female individuals (*F*_8,18_ = 48.674, *p* ≤ 0.001) (Figure 2A). Further analysis found that the expression of *PxVMP26* started at the third day of the pupal stage and reached its maximum value at the second day of adult emergence (Figure 2B) (*F*_11,24_ = 98.464, *p* ≤ 0.0001). Tissue-specific expression profiles showed that *PxVMP26* was highly expressed in ovary. The expression level in the ovarian tubes with incomplete yolk deposition was significantly higher than that in the ovarian tubes with complete yolk deposition (*F*_7,16_ = 22.532, *p* ≤ 0.001) (Figure 2C). Western blot analysis revealed a similar expression profile of *Px*VMP26 protein as its transcript detection (Figure 2D). 

### 2.3. The mutation of PxVMP26 Produced by CRISPR/Cas9 

**Figure 3:**
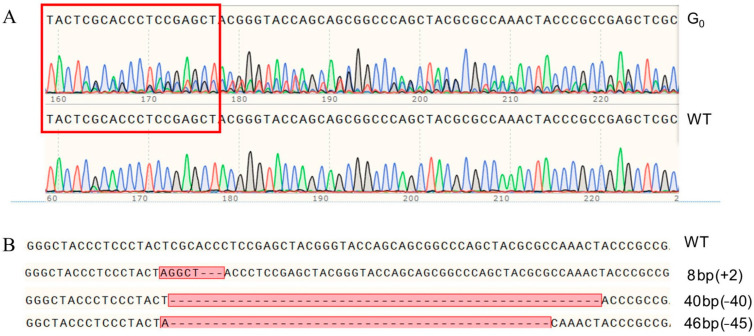
Sequencing and identification of the mutant genotypes of *PxVMP26* based on CRISPR/Cas9. (**A**) Representative sequencing maps of PCR products from wild type WT and G_0_ adults with mutation at the target site, which are highlighted in the red boxes. (**B**) The mutant types of *PxVMP26* gene in G_1_ generation. The mutant sites are indicated by the red boxes. The deleted bases are displayed with dashed lines, and the inserted bases are shown in the red boxes. The numbers of mutant bases are demonstrated at the right of each allele (–, deletion; +, insertion). Eight bp mutation, 5-base insertion and 3-base deletion; 40 bp mutation, 40-base deletion; 46 bp mutation, 1-base insertion and 45-base deletion.

A total of 52 preblastoderm eggs of *P. xylostella* were microinjected with sgRNA and Cas9 mixture, with 19.2% (10/52) of these eggs hatching. Among them, 90% (9/10) of these individuals successfully developed into adult stage and were defined as the G0 generation. Sequencing analysis of all G0 individuals showed a mutation efficiency of 22.2% (2/9) in the target site of *PxVMP26* gene, as indicated by the multi-peaks at the target site in the sequencing chromatogram (Figure 3A).

Each virgin G1 adult produced by mutant parental G0 was randomly mated with a newly emerged WT adult to produce the G2 offspring. The mutation rate was 79.2% (19/24) in G1 adults. All mutant G1 individuals were further genotyped, and three mutant types were identified, including one with 8 bp (5 bp insertion and 3 bp deletion), one 40 bp deletion, and one with 46 bp (1 bp insertion and 45 bp deletion) (Figure 3B). 

### 2.4. Establishment of the Homozygous PxVMP26 Mutant Lines

**Figure 4:**
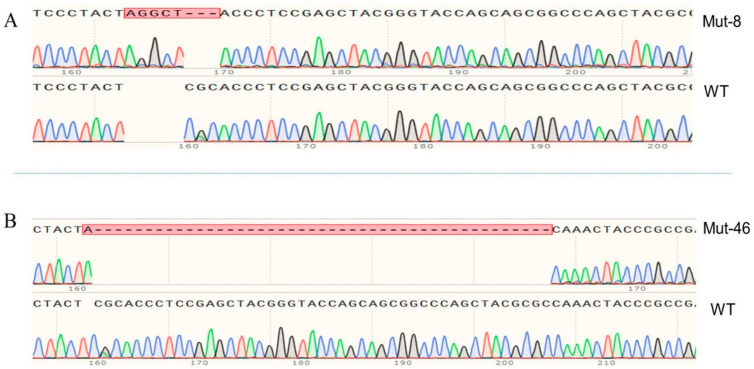
Homozygous mutation types of the *PxVMP26* gene. (**A**) Eight bp mutation (5-base insertion and 3-base deletion) and (**B**) 46 bp mutation (1-base insertion and 45-base deletion) are highlighted with red box.

The offspring produced by the parents (G1) with the mutations of 8 and 46 bp were retained as G2 to develop the sib-cross strains. In the 8 bp mutation, the sib-cross pairs with single homozygotes (aa^♀^ × Aa^♂^/aa^♂^ × Aa^♀^) were obtained in G3, of which offspring were continuously sib-crossed, and the double homozygotes (aa^♀^ × aa^♂^) were obtained in G4 in 13.3% (2/15) of pairs. The propagation continued until the stable homozygous mutant strain in G12 (Figure 4A). In the 46 bp mutation, the single homozygotes (aa^♀^ × Aa^♂^/aa^♂^ × Aa^♀^) were obtained in G5, and the double homozygotes (aa^♀^ × aa^♂^) were obtained in G7 with 7.4% (2/27) of the pairs. The stable homozygous mutant strain was established in G13 (Figure 4B).

**Figure 5:**
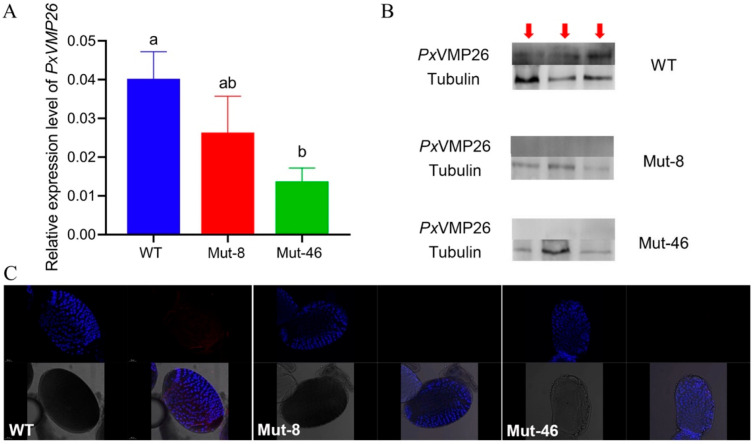
The mutation efficiency of *PxVMP26* induced by CRISPR/Cas9. (**A**) The transcription levels of *PxVMP26* were analyzed by qRT-PCR; the mRNA level was normalized to ribosomal protein L32 (*RIBP*). Data shown as mean ± SE represented with three biological replicates. Different letters on the bars indicate significant differences (*p* < 0.05) using LSD’s multiple range test. (**B**) The expression profiles of *Px*VMP26 protein were analyzed by western blot; 30 ug of protein was separated using 15% SDS-PAGE, and tubulin was used as the internal reference. The VMP26 protein is indicated by the red arrows. (**C**) The freshly dissected ovaries from newly emerged Mut and WT females were treated with the *Px*VMP26 polyclonal antibody and Alex Fluor Plus 594-conjugated secondary antibody (goat anti-rabbit) (red) and stained with DAPI Fluoromout-G^TM^ for DNA (blue); bar = 50 um.

The mutation efficiency was evaluated based on mRNA expression, protein content, and immunoreactivity. qPCR analysis showed that there was no significant difference in the expression level of the *PxVMP26* gene between WT and Mut-8 female adults (*F*_2,6_ = 10.588, *p* = 0.053), as well as between Mut-8 and Mut-46 female adults (*F*_2,6_ = 10.588, *p* = 0.710). However, the expression of the *PxVMP26* gene in female adults of Mut-46 was significantly reduced compared with that of WT (*F*_2,6_ = 10.588, *p* = 0.004) (Figure 5A). No *Px*VMP26 protein was detected in female adults of Mut-8 and Mut-46 strains using western blot analysis (Figure 5B), as well as in the ovaries using immunofluorescence (Figure 5C).

### 2.5. Effects of PxVMP26 Knockout on the Fertility of P. xylostella

**Figure 6:**
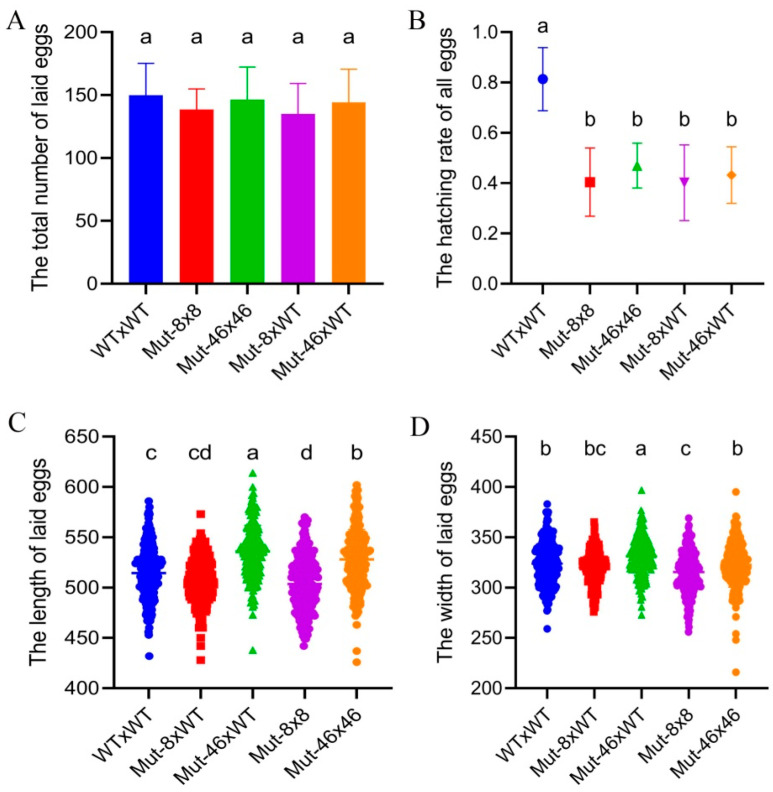
The fecundity and egg size of *P. xylostella* after *PxVMP26* knockout. (**A**) The total number of laid egg. (**B**) Hatching rate. (**C**) Egg length. (**D**) Egg width. Data shown as mean ± SE. Different letters on the bars indicated significant differences (*p* < 0.05) using LSD’s multiple range test. WT × WT, WT^♀^ mated with WT^♂^; Mut-8 × 8, Mut-8^♀^ mated with Mut-8^♂^; Mut-46 × 46, Mut-46^♀^ mated with Mut-46^♂^; Mut-8 × WT, Mut-8^♀^ mated with WT^♂^; Mut-46 × WT, Mut-46^♀^ mated with WT^♂^.

The mean numbers of eggs laid per female from different mating treatments, including WT × WT, Mut-8 × 8, Mut-46 × 46, Mut-8 × WT, and Mut-46 × WT, were 149.9, 138.5, 146.4, 135.1, and 144.3, respectively. No significant difference was detected (*F*_4,115_ = 1.494, *p* > 0.05) (Figure 6A). The hatching rate dropped sharply from 81% for WT × WT to 40% for Mut-8 × 8, 47% for Mut-46 × 46, 40% for Mut-8 × WT, and 46% for Mut-46 × WT (*F*_4,115_ = 47.523, *p* < 0.05). No significant difference was found between the mating treatments of Mut-8 and Mut-46 (*F*_4,115_ = 47.523, *p* > 0.05) (Figure 6B).

The mean length and width of eggs significantly decreased from 514.5 and 323.8 μm for WT × WT to 503.7 (*F*_4,1145_ = 61.066, *p* < 0.01) and 315.6 μm (*F*_4,1145_ = 19.065, *p* < 0.01) for Mut-8 × 8. There was no obvious difference between WT × WT and Mut-8 × WT (length, *F*_4,115_ = 61.066, *p* > 0.05; width, *F*_4,1145_ = 19.065, *p* > 0.05) (Figure 6C,D). The mean size of the eggs produced by Mut-46 × WT was 535.9 μm in length and 331.1 μm in width, which were larger than those produced by WT × WT (length: *F*_4,1145_ = 61.066, *p* < 0.01; width: *F*_4,1145_ = 19.065, *p* < 0.01). The mean egg length of Mut-46 × 46 was 528.1 μm, which was significantly longer than that of WT × WT, but the mean egg width was not significantly different between Mut-46 × 46 and WT × WT (Figure 6C,D).

### 2.6. Effects of PxVMP26 Knockout on Vitelline Deposition and Water Retention of P. xylostella Eggs

**Figure 7:**
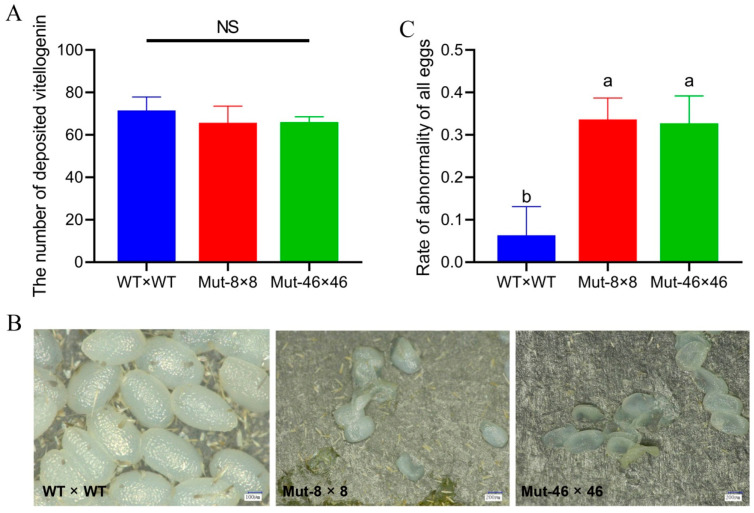
(**A**) The vitelline deposition of *P. xylostella* eggs after *PxVMP26* knockout. WT × WT, WT^♀^ mated with WT^♂^; Mut-8 × 8, Mut-8^♀^ mated with Mut-8^♂^; Mut-46 × 46, Mut-46^♀^ mated with Mut-46^♂^. (**B**) Morphologies of eggs were observed by digital microscope VHX-2000C (KEYENCE, Japan), bar = 100 μm in WT and bar = 200 μm in Mut. (**C**) Rate of abnormality of the eggs. Data shown as mean ± SE. NS indicates no significant difference (*p* > 0.05), and different letters on the bars indicate significant differences (*p* < 0.05), using LSD’s multiple range test.

The numbers of eggs with complete vitelline deposition in the ovarian tubes did not differ (*F*_2,27_ = 3.451, *p* > 0.05) with values of 71.5 for WT, 63.7 for Mut-8, and 66.0 for Mut-46 (Figure 7A). The *PxVMP26* knockout females laid more fragile eggs, most of which collapsed after 48 h after oviposition. Abnormality rates due to dehydration significantly increased from 5% for WT to 43% and 34% for Mut-8 and Mut-46 (*F*_2,30_ = 42.315, *p* < 0.05) (Figure 7B,C). There was no significant difference between the two different mutant lines (*F*_2,30_ = 42.315, *p* > 0.05) (Figure 7B,C).

### 2.7. Effects of PxVMP26 Knockout on the Formation of Vitelline Membrane

**Figure 8:**
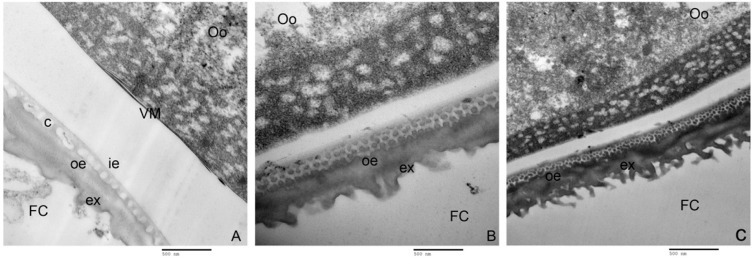
Microscopic structure of the *P. xylostella* vitelline membrane after *PxVMP26* knockout. (**A**) WT. (**B**) Mut-8. (**C**) Mut-46. The ultrastructure of vitelline membrane was observed by transmission electron microscope (TME) (H-7650, HITACHI), bar = 500 nm. Oo, oocyte; FC, follicular cell; VM, vitelline membrane (electron-dense region); ex, exochorion; ie, inner-endochorion; oe, outer-endochorion; c, columnar layer.

Ultrathin sections of ovarian follicles from WT and Mut lines, observed with TEM, indicated that *PxVMP26* knockout effectively inhibited the formation of the vitelline membrane and the deposition of endochorion layers, but did not affect the exochorion formation (Figure 8). In WT, the oocytes were closely enclosed by the vitelline membrane, and the column layer between the inner- and outer-endochorion could be clearly seen (Figure 8A). However, no obvious vitelline membrane was observed on the surface of the oocytes from Mut-8 and Mut-46. The endochorion was also not found in Mut-8 and Mut-46, and the column layer between the inner- and outer-endochorion was replaced by the sponge-like structure (Figure 8B,C).

## 3. Discussion

The eggshell is a complex ultrastructure, which is formed by some structural and non-structural proteins secreted by follicular cells in a certain order of time and space [7]. The vitelline membrane, as the main inner eggshell structure, along with the outer eggshell wrap around the egg and act as water-proofing and protection from environmental damage [41]. Our study contributed to the exploration, through the use of the CRISPR/Cas9 system, of the functions of the key structural protein VMP of the inner eggshell regarding egg formation and embryonic development in *P. xylostella*.

The three *VMP* genes identified from the *P. xylostella* genome [31] and ovary transcriptome [32] were highly specifically expressed in the *P. xylostella* ovary. According to the theoretical molecular weight of ~26 kDa, it was renamed as vitelline membrane protein 26 (*PxVMP26*) [42]. *Px*VMP26 consisted of a single exon, i.e., a non-secretory protein with no signal peptide. It was similar to the predicted characteristics of VMP23 in *B. mori* [26]. *Px*VMP26 was rich in leucine, serine, alanine, and proline, just like the small molecular VMPs of other insects. Many studies have reported that the dipteran and the lepidopteran VMPs contain high levels of leucine, alanine, proline, and hydrophilic serine residues (Elalayli et al., 2008; Xu et al., 2012; Xu 2013). 

No VM domain was found in *Px*VMP26. Papantonis et al. reported that *Drosophila* VMPs are small molecular proteins with a conserved hydrophobic C terminus (VM domain) [13]. In *B. mori*, only three of the five putative VMP proteins have a recognizable VM domain [26,27]. These results suggest that the structural characteristics of VMP are species-specific. Xu also show that, compared with the consistency of Diptera VMPs, the Lepidopteran VMPs greatly vary in terms of the occurrences of introns, the VM domain, and macromolecule VMPs, and amino acid composition [42]. The evolutionary rate of the genome in Lepidoptera may be faster than that of other insect species [43]. Therefore, we speculated that the diversity of VMPs in Lepidoptera might be due to their rapid evolution. 

Both qPCR and western blot analyses indicated that the *Px*VMP26 gene was only expressed in female individuals, and its transcription began on the third day of the pupal stage and reached the highest level in the adult stage. This almost synchronous expression pattern with the ovary development and vitellogenesis of *P. xylostella* implied that *PxVMP26* transcription might occur during the vitellogenesis. The expression of *PxVMP26* was highest in the ovaries and especially in the ovarian tubes with incomplete yolk deposition, further supporting the importance of *PxVMP26* during vitellogenesis in *P. xylostella*. In most insects, VMPs are synthesized by the follicular epithelium during vitellogenesis and form functional proteins just prior to choriogenesis [25,44,45]. In *B. mori,* the *VMPs* (*VMP23*, *VMP25*, *VMP30*, *VMP30.1*, *VMP90*, and *EP80*) are preferentially expressed in the ovary [26,27,46]. All *VMPs*, except *EP80* and *VMP25*, are nearly consistently expressed during vitellogenesis in follicles, first appearing in early vitellogenic follicles, accumulating in the follicles during middle to late vitellogenesis, and disappearing in early choriogenesis [26,27]. In *D. melanogaster*, *VMP* gene transcription is confined to females during vitellogenesis [47]. In *Z. cucurbitae*, *VMP26s* are highly expressed in vitellogenic adult females, mainly in the ovaries [18,48]. Vmp26Aa protein in *B. dorsalis* is found in high abundance in the middle vitellogenic follicles [17]. These studies demonstrate the crucial role of *VMPs* in the reproduction of insects.

The CRISPR/Cas9 system has contributed to the efficient investigation of the gene functions in insects, especially in non-model agricultural pests where effective RNAi is hard to achieve, such as in Lepidoptera [39,40,49]. Here, CRISPR/Cas9-mediated *PxVMP26* knockout successfully built two homozygous strains with 8- and 46-base pair mutations. However, only one of two co-injected sgRNAs caused the mutation in G0 generation, presumably because the co-injected sgRNAs were too close to each other. Zhang and Reed (2017) reported that the mutation efficiency of CRISPR/Cas9 gene editing may be related to the distance between the co-injected sgRNAs, sgRNA mixture concentration, or injection procedure [50]. 

After the knockout of *PxVMP26*, although there was no difference in expression levels between the two mutant lines, the transcriptional level of the *PxVMP26* gene was effectively suppressed in Mut-46 female adults, but the expression in Mut-8 was not reduced. In general, Cas9-mediated knockout affects mRNA translation and has no effect on protein expression [39,51]. As one of the most important biological processes in cells, mRNA translation is subject to much quality control (QC) [52]. Two QC pathways involved in CRISPR/Cas9-mediated mRNA degradation in gene knockout, including nonsense-mediated mRNA decay and no-stop decay, might have been at play in this experiment [37,52,53].

Insect eggshells are responsible for maintaining permeability during embryogenesis to avoid water loss [4,26]. In our experiment, the eggs from Mut-8 and Mut-46 were more fragile and more likely to collapse, while the abnormality rates significantly increased and the hatching rates sharply dropped. A deficit of *PxVMP26* suppressed the vitelline membrane formation and the inner-endochorion layer deposition. Similar effects have also been reported for other insects. Xu et al. showed that the knockdown of *BmEP80* can disrupt eggshell permeability, leading to water loss, collapse, and death. Chen et al. indicated that the loss of *EP80* affects the structural integrity of inner eggshell (vitelline membrane) and water retention in *B. mori* eggs, resulting in a non-viable phenotype [28]. In *B. mori*, inhibition of the transcription of *BmVMP30* and *BmVMP90* damages the follicular epithelium integrity, and disrupts the endochorion deposition [25,27]. Manogaran and Waring showed that *VMP26Ab* mutant *D. melanogaster* produces infertile eggs with collapsed eggshells [20]. The knockout of *VMP26Ab* in *Z. cucurbitae* leads to defects in the eggshell structure, and results in rapid water loss during oogenesis, as well as increased egg abnormality and decreased hatchability [19].

Here, no significant difference in vitelline deposition and oviposition between the Mut and WT strains was found, suggesting that the lack of the vitelline membrane may not affect vitellogenic development and ovulation in *P. xylostella*. In *B. mori*, similar observations are reported with a deficiency in *BmVMP30* still leading to complete vitellogenic development and choriogenesis [25]. Although the follicular epithelial tissue is compromised due to *VMP* loss in *B. mori*, the program of chorion gene expression remains intact [13,27]. Lou et al. indicated that ovulation begins when the choriogenesis is completed in *Nilaparvata lugens* [5]. Other studies have shown that some insects are still able to lay eggs as long as the chorion layer is fully formed despite lacking vitellogenin [39,40,54]. Here, the absence of *PxVMP26* had no significant effect on the structure of exochorion on *P. xylostella* oocyte. Thus, we speculated that *PxVMP26* might be necessary for the integrity of the inner eggshell, especially through the vitelline membrane formation and the inner-endochorion layer deposition, for water retention.

## 4. Materials and Methods

### 4.1. Insect Colony 

The insecticide susceptible *P. xylostella* colony Geneva 88 (hereafter G88) was obtained from Professor Shelton at Cornell University (October 2016) and has since been maintained in climate chamber for more than 46 generations at the Fujian Agriculture and Forestry University. Larvae were fed on the fresh artificial diet without any toxin in Dixie paper cups (10.4 cm × 7.3 cm × 8.5 cm), and pupae were transferred into a new cup until hatching. Newly emerged adults were fed with 10% honey solution for nutrition [39,40]. The rearing condition was 25 ± 2 °C, L:D = 16:8 photoperiod, and 70–80% relative humidity. 

### 4.2. Total RNA Extraction and cDNA Synthesis 

Total RNA was extracted from individuals or tissues (head, thorax, midgut, fat body, ovary, epidermis, and ovarian tube with complete and incomplete yolk deposition) with the Eastep^®^ Super Total RNA Extraction Kit (Promega, Madison, WI, USA). The RNA concentration and quality were measured with the Nano Vue spectrophotometer (GE-Healthcare, Little Chalfont, UK), followed by a 1% agarose gel electrophoresis for RNA integrity. The cDNA was synthesized using the Hiscript^TM^ Reverse Transcriptase (Vazyme, Nanjing, China) with RNA concentration of 500 ng/µL.

### 4.3. PxVMP26 Cloning

The *VMP26* sequences obtained from the *P. xylostella* Genome Database (http://59.79.254.1/DBM/index.php, accessed on 19 December 2014) and transcriptome data [32] were identified by common PCR using specific primers designed with SnapGene 2.3.2 (Table 1). The PCR reaction was performed as follows: 94 °C, 3 min for pre-degeneration, then 94 °C, 30 s, 60 °C, 30 s, 72 °C, 52 s for 34 cycles, and finally 72 °C, 5 min for an extension. All PCR products were purified and linked with the pJET1.2 vector (Thermo Fisher Scientific, Waltham, MA, USA) for sequencing. 

### 4.4. Sequence Comparison and Phylogenetic Analysis of PxVMP26

The amino acid sequence of *Px*VMP26 was deduced with the translation tool in SnapGene 2.3.2 (GSL Biotech LLC, San Diego, CA, USA). Its sequence similarity with other known lepidopteran VMPs, which were downloaded from the protein database of the National Center for Biotechnology Information (NCBI), was compared using BLAST program in BioEdit 7.1.9 (Borland, Scotts Valley, CA, USA). The homologous VMP sequences were aligned by Clustal X 2.0 [55], and the phylogenetic tree was constructed with MEGA 7 through neighbor joining method. Bootstrap test (100 replicates) was used to estimate the confidence values.

### 4.5. Expression Patterns of PxVMP26

#### 4.5.1. Sample Preparation

For stage- and sex-specific expression patterns, samples from different stages and sexes (eggs, 1–4 instar larvae, 1–3 d female and male pupae, and 1–3 d female and male adults) were collected and stored at −80 °C [39,40]. For tissue-specific expression patterns, newly emerged adults were dissected to extract the head, thorax, midgut, fat body, ovary, epidermis, and ovarian tube with complete and incomplete yolk deposition in DNase and RNase free water (QIAGEN, Dusseldorf, Germany). Each tissue was separately placed in 1× phosphate-buffered saline (PBS) and stored at −80 °C. Each sample consisted of three biological replicates.

#### 4.5.2. qRT-PCR 

RNA extraction and cDNA synthesis of each sample was conducted as described in Section 2.2. The qRT-PCR was performed using the GoTag^®^ qPCR Master Mix Kit (Promega, USA) as follows: 95 °C, 10 min; then 95 °C, 15 s, and 60 °C, 60 s for 40 cycles; 90 °C, 15 s, 60 °C, 60 s, and 95 °C, 15 s. Ribosomal protein genes L32 (RPL32), L8 (RPL8), and elongation factor 1 alpha (EF-1α) were used as the internal references to normalize the transcript levels according to the comparative Ct method (2^−∆Ct^). Specific primers used for this reaction are listed in Table 1.

#### 4.5.3. Protein Preparation and Western Blot

A peptide sequence (CSTGDVELKGFKDIV) at the C-terminal of *Px*VMP26 was selected to be artificially synthesized, conjugated with cysteine residue, and used as an antigen to inject rabbits for polyclonal antibody production at GenScript Biotech Corp. (Nanjing, China).

Total proteins from whole bodies or tissues of *P. xylostella* were extracted according to Wang et al. [56]. Each protein sample was dissolved in a buffer (8 M Urea, 1% SDS in pH 8.0 Tris-HCl) at 50 °C, and protein contents were measured with BCA protein Quantification Kit (Yeasen, Shanghai, China). For western blot, the same amounts of total proteins from different stages (30 μg) or tissues (20 μg) were separated on 15% SDS-PAGE using a Mini protein system (Bio-Rad, Hercules, CA, USA), and then transferred onto a polyvinylidene difluoride membrane (Millipore, Billerica, MA, USA) under 180 mA electric current. The transfer time of reference and target protein was 40 and 20 min, respectively. The membrane was then blocked with 3% (*w*/*v*) BSA in 1×TBST and overnight at 4 °C. It was then incubated with tubulin antibody (1:1000) (Sigma-Aldrich, St. Louis, MO, USA) and *Px*VMP26 primary antibody (1:1000) at room temperature for 2 h and washed three times with 1×TBST for 10 min at a time. Finally, the membrane was incubated again with the secondary antibody (1:5000) of Goat Anti-Rabbit IgG-HRP (Boster, Wuhan, China) for 1 h at room temperature and washed as described above. Chromogenic reaction was detected using the Immun-Star Western C chemiluminescence detection kit (Bio-Rad, Hercules, CA, USA) and photographed by Fusion Fx system (VILBER BIO IMAGING, Paris, French) [39,40].

### 4.6. Immunofluorescence Analysis

The freshly dissected ovaries of *P. xylostella* were rinsed with PBS, then fixed with 4% paraformaldehyde at room temperature for 30 min, and finally rinsed with PBS 3 times. Each sample was soaked with 0.1% Triton X-100 at room temperature for 30 min and rinsed with 1×TBST 5 times. Then, each sample was blocked with 3% BSA (1.5 g BSA volume to 50 mL 1×TBST) at room temperature for 1 h, and incubated with the *Px*VMP26 antibody (1:200) at room temperature for 2 h or at 4 °C overnight. The samples were rinsed with 1×TBST 10 times. Subsequently, each sample was incubated with Alex Fluor Plus 594-conjugated secondary antibody (1:200) (goat anti-rabbit, Thermo Fisher Scientific, Waltham, MA, USA) at room temperature in dark for 2 h or at 4 °C overnight and rinsed 10 times in 1×TBST, and dyed with DAPI Fluoromout-GTM staining (Em = 455 nm, Yeasen, Shanghai, China). Fluorescence reaction was observed and photographed with the Leica SP5 confocal laser-scanning microscope (Leica, Wetzlar, Germany) at 405–561 nm wavelength.

### 4.7. sgRNA Design and Synthesis 

The 292–311 bp and 320–339 bp regions of *PxVMP26* gene were selected as the target sites, and CRISPR gRNA Design tool (https://www.atum.bio/eCommerce/cas9/input, accessed on 2 February 2021) was used to design sgRNAs and evaluate their off-target effects (Figure S1). Two oligonucleotides primers were used in the PCR reaction by the PrimeSTAR^®^ HS DNA Polymerase to prepare the transcription template for in vitro sgRNA synthesis [49] (Table 1). PCR products were purified by universal DNA purification kit (TIANGEN, Beijing, China), and their concentration and purity were detected by NanoVue spectrophotometer (GE-Healthcare) and agarose gel electrophoresis, respectively. In vitro sgRNA synthesis was performed using HiScribe T7 Quick High Yield RNA Synthesis Kit (NEB, Ipswich, MA, USA) following the manufacturer’s instructions.

### 4.8. sgRNA/Cas9 Protein Microinjection 

Fresh eggs at preblastodermal stage were collected with 10 cm^2^ parafilm sheets precoated with cabbage leaf extract, and each sheet was replaced with a new one every 15 min, according to Huang et al. [49] and Zou et al. [40]. To form a stable ribonucleoprotein complex, 250 ng sgRNA_1_/sgRNA_2_ were mixed with 1500 ng Cas9 protein (GenScript, China) and incubated at 37 °C for 10 min. The solution was then injected into the eggs posterior pole using an Olympus SZX16 microinjection system (Olympus, Japan) within 15 min of oviposition. The injected eggs were carefully placed in 90 mm Petri dishes with a filter paper moistened with sterile water, and maintained at 25 ± 1 °C, 60–70% RH in dark environment for hatching. Larvae were transferred to the Dixie cups and fed according to the feeding method described in Section 2.1.

### 4.9. Genetic Crosses and PCR-Based Genotyping

The stable homozygous mutant strains of *PxVMP26* gene were established with a serial crossing scheme. The eggs microinjected with sgRNA and Cas9 mixture were developed to adults as the initial generation 0 (G0). Virgin G0 adults were single mated with virgin wild types (WT) adults to produce a transgenic line (F1), and then each G0 individual was genotyped through sequencing. Virgin F1 adults produced by the mutant G0 adults were outcrossed with virgin WT adults to generate G2 offspring. All F1 adults were then individually identified by PCR-based genotyping to verify the mutations. F2 individuals produced by heterozygous F1 with the same allelic mutation were intercrossed to produce F3 progeny. Those individuals produced by the homozygous mutation parents (F2) were kept to develop the stable homozygous lines of *PxVMP26* (MUT). Intercrossing continued until homozygous mutations were generated [39,40].

Genomic DNA (gDNA) of individual adults was isolated with TIANamp Genomic DNA Kit (TIANGEN, China) according to the manufacturer’s instructions. The gDNA fragments of *PxVMP26* (500 bp) were amplified with the specific primers listed in Table 1 to detect the mutations in the target region. The PCR procedure was as follows: one cycle of 94 °C for 3 min, and 34 cycles of 94 °C for 30 s, 60 °C for 30 s, 72 °C for 30 s, and one cycle of 72 °C for 5 min. Subsequently, the PCR products of F1 individuals with mutations were purified using the Gel Extraction Kit (Omega, USA) and linked with the pJET1.2/blunt vector (Thermo, China) for sequencing to verify the mutant types (insertion or deletion).

### 4.10. Phenotypic Observation and Bioassays

Ten ovaries were dissected from Mut and WT females after 48 h of emergence as previously described in Section 2.5 and rinsed with PBS 3 times. The number of fully developed oocytes were recorded using digital microscope VHX-2000C (KEYENCE, Osaka, Japan).

Each single pair of newly emerged adults was placed in a Dixie cup with a parafilm sheet containing the cabbage leaf extract for egg laying and 10% honey solution cotton ball for nutrition. Twenty-four pairs of *P. xylostella* adults were used for each treatment. The cups were kept at 25 ± 1 °C, photoperiod L:D = 16:8 and 60–70% relative humidity. Each sheet was removed and replaced with a new one every day. The length and width of the eggs were measured by digital microscope VHX-2000C (KEYENCE, Japan) (n = 230 for each treatment). The total number of eggs laid in 3 days (most eggs are laid in the first three days, reference) and the hatching rate of eggs laid by each pair were recorded. The abnormality rate of eggs laid by eleven pairs of newly emerged *P. xylostella* adults was calculated after 48 h of oviposition [19].

### 4.11. Microstructure Observation of Vitelline Membrane

The ovaries of Mut and WT females after 48 h of emergence were dissected. Within each ovary, eggs with complete yolk deposition were collected and placed at −80 °C for 12 h. The samples were fixed in 2.5% glutaraldehyde in PBS for 24 h, and the contents were extruded using a sterile grinding rod, and centrifuged at 12,000 rpm for 2 min. The supernatant was removed, and the precipitates were fixed in 200 μL glutaraldehyde for 24 h. The newly obtained samples were washed with 0.1 M pH 7.0 1×PBS 3 times and fixed with 1% osmium tetroxide in PBS for 1.5 h at room temperature. The fixed samples were washed again with 0.1 M pH 7.0 1×PBS 3 times, and dehydrated in grade series of ethanol up to 100%, followed in 100% ethanol and 100% acetone (1:1), and 100% acetone for 30 min. Subsequently, the sample was embedded in Spurr’s resin (SPI Ltd., Suzhou, Jiangsu, China) and polymerized at 70 °C for 24 h. Sections were stained with both uranyl acetate and lead (II) citrate trihydrate to observe the effects of *PxVMP26* knockout on the ultrastructure of vitelline membrane by transmission electron microscope (TME) (H-7650, HITACHI, Japan). 

### 4.12. Statistical Analyses

Multiple comparisons were conducted by one-way analysis of variance (ANOVA) using LSD’s multiple range test, which were completed by SPSS 25.0 software (SPSS Inc., Chicago, IL, USA). Data of the hatching rate were arcsine-transformed to satisfy the assumption of normality.

## 5. Conclusions

In conclusion, we successfully knocked out *PxVMP26* using the CRISPR/Cas9 system and clarified its roles in the oocyte formation and embryonic development of *P. xylostella*. These results suggest that *PxVMP26* is indispensable for eggshell formation, thus critical for embryonic development in *P. xylostella*. This is the first study to efficiently explore the reproductive regulation of *PxVMP26* in *P. xylostella* using CRISPR/Cas9 technology. Further work needs to be carried out to confirm whether *PxVMP26* plays a role in embryogenesis, especially in the formation and differentiation of syncytial and cellular blastoderms, as well as to assess the potential of the *PxVMP26* gene in controlling *P. xylostella* and other agriculture pests by siRNA- or sgRNA-mediated silencing or knockout of the target gene.

## Figures and Tables

**Table 1:** Primers used in this study.

Purpose	Primer Name	Primer Sequence 5′-3′	Position
Common PCR	C-VMP26 F	ATGGTGCCCCTGGCGGAG	1–18
	C-VMP26 R	TCAGTAGCCATATCCAGGCCTTGG	829–852
qRT-PCR	Q-RPL32 F	CAATCAGGCCAATTTACCGC	5–24
	Q-RPL32 R	CTGCGTTTACGCCAGTTACG	94–113
	Q-RPL8 F	CGGTCGTGCCTACCACAAATACA	560–582
	Q-RPL8 R	CGTGAGGATGCTCCACAGGGT	628–648
	Q-EF-1α F	GCCTCCCTACAGCGAATC	477–494
	Q-EF-1α R	CGTGAGGATGCTCCACAGGGT	620–637
	Q-VMP26 F	CGATGCAGCCGCTGGAAGA	104–122
	Q- VMP26 R	GTAGTCGTAGTCCCTCGCAG	266–285
sgRNA synthesis	sgRNA-F1 *	TAATACGACTCACTATA TAGCCCGCGTATCCCGGGCT **GTTTTAGAGCTAGAAATAGCAAGTTAAAATAAGGCTAGTCC**	-
	sgRNA-F2 *	TAATACGACTCACTATA GTAGCTCGGAGGGTGCGAGT **GTTTTAGAGCTAGAAATAGCAAGTTAAAATAAGGCTAGTCC**	
	sgRNA-ComR ^#^	AAAAGCACCGACTCGGTGCCACTTTTTCAAGTTGATAACGGACTAGCCTTATTTTAACTTGCTATTTCTAGCTCTAAAA	
Genotyping	VMP26-F	ACATGGACAACGTGCTACCG	131–150
	VMP26-R	TTTGAGTTCTACGTCGCCCG	611–630

* T7 promoter sequence is underlined, and sgRNA skeleton sequence is in bold. ^#^ It is a universal reverse primer.

## Data Availability

The data presented in this study are available from the corresponding author upon reasonable request.

## Supplementary Materials

The supporting information can be downloaded at: https://www.mdpi.com/article/10.3390/ijms23179538/s1.

## References

[1] Woods H.A., Bonnecaze R.T., Zrubek B. (2005). Oxygen and water flux across eggshells of *Manduca sexta*. J. Exp. Biol..

[2] Margaritis L.H., Gilbert L.I., Kercut G.A. (1985). Structure and physiology of the eggshell. Comprehensive Insect Physiology, Biochemistry and Pharmacology.

[3] Irles P., Piulachs M.D. (2011). Citrus, a key insect eggshell protein. Insect Biochem. Mol. Biol..

[4] Woods H.A. (2010). Water loss and gas exchange by eggs of *Manduca* sexta: Trading off costs and benefits. J. Insect Physiol..

[5] Lou Y.H., Pan P.L., Ye Y.X., Cheng C., Xu H.J., Zhang C.X. (2018). Identification and functional analysis of a novel chorion protein essential for egg maturation in the brown planthopper. Insect Mol. Biol..

[6] Furriols M., Casanova J. (2014). Germline and somatic vitelline proteins colocalize in aggregates in the follicular epithelium of *Drosophila* ovaries. Fly.

[7] Velentzas A.D., Velentzas P.D., Katarachia S.A., Anagnostopoulos A.K., Sagioglou N.E., Thanou E.V., Tsioka M.M., Mpakou V.E., Kollia Z., Gavriil V.E. (2018). The indispensable contribution of s38 protein to ovarian-eggshell morphogenesis in *Drosophila melanogaster*. Sci. Rep..

[8] Zrubek B., Woods H.A. (2006). Insect eggs exert rapid control over an oxygen–water tradeoff. Proc. Biol. Sci..

[9] Isoe J., Koch L.E., Isoe Y.E., Rascon A.A., Brown H.E., Massani B.B., Miesfeld R.L. (2019). Identification and characterization of a mosquito-specific eggshell organizing factor in *Aedes aegypti* mosquitoes. PLoS Biol..

[10] Mineo A., Furriols M., Casanova J. (2015). Accumulation of the *Drosophila* Torso-like protein at the blastoderm plasma membrane suggests that it translocates from the eggshell. Development.

[11] Taylor S.E., Tuffery J., Bakopoulos D., Lequeux S., Warr C.G., Johnson T.K., Dearden P.K. (2019). The torso-like gene functions to maintain the structure of the vitelline membrane in *Nasonia vitripennis*, implying its co-option into *Drosophila* axis formation. Biol. Open.

[12] Dos Santos G., Schroeder A.J., Goodman J.L., Strelets V.B., Crosby M.A., Thurmond J., Emmert D.B., Gelbart W.M., FlyBase C. (2015). FlyBase: Introduction of the *Drosophila melanogaster* Release 6 reference genome assembly and large-scale migration of genome annotations. Nucleic Acids Res..

[13] Papantonis A., Swevers L., Iatrou K. (2015). Chorion genes: A landscape of their evolution, structure, and regulation. Annu. Rev. Entomol..

[14] Marinotti O., Ngo T., Kojin B.B., Chou S.P., Nguyen B., Juhn J., Carballar-Lejarazú R., Marinotti P.N., Jiang X., Walter M.F. (2014). Integrated proteomic and transcriptomic analysis of the *Aedes aegypti* eggshell. BMC Dev. Biol..

[15] Amenya D.A., Chou W., Li J., Yan G., Gershon P.D., James A.A., Marinotti O. (2010). Proteomics reveals novel components of the *Anopheles gambiae* eggshell. J. Insect Physiol..

[16] Gonçalves V.R., Jr Sobrinho I.S., Jr Malagó W., Henrique-Silva F., de Brito R.A. (2013). Transcriptome analysis of female reproductive tissues of *Anastrepha obliqua* and molecular evolution of eggshell proteins in the *fraterculus* group. Insect Mol. Biol..

[17] Wei D., Li R., Zhang M.Y., Liu Y.W., Zhang Z., Smagghe G., Wang J.J. (2017). Comparative proteomic profiling reveals molecular characteristics associated with oogenesis and oocyte maturation during ovarian development of *Bactrocera dorsalis* (Hendel). Int. J. Mol. Sci..

[18] Wei D., Zhang Y.X., Liu Y.W., Li W.J., Chen Z.X., Smagghe G., Wang J.J. (2019). Gene expression profiling of ovary identified eggshell proteins regulated by 20-hydroxyecdysone in *Bactrocera dorsalis*. Comp. Biochem. Physiol. Part D Genom. Proteom..

[19] Li W., Song Y., Xu H.J., Wei D., Wang J. (2021). Vitelline membrane protein gene *ZcVMP26Ab* and its role in preventing water loss in *Zeugodacus cucurbitae* (Coquillett) embryos. Entomol. Gen..

[20] Manogaran A., Waring G.L. (2004). The N-terminal prodomain of sV23 is essential for the assembly of a functional vitelline membrane network in *Drosophila*. Dev. Biol..

[21] Fakhouri M., Elalayli M., Sherling D., Hall J.D., Miller E., Sun X., Wells L., LeMosy E.K. (2006). Minor proteins and enzymes of the *Drosophila* eggshell matrix. Dev. Biol..

[22] Wu T., Manogaran A.L., Beauchamp J.M., Waring G.L. (2010). *Drosophila* vitelline membrane assembly: A critical role for an evolutionarily conserved cysteine in the “VM domain” of sV23. Dev. Biol..

[23] Elalayli M., Hall J.D., Fakhouri M., Neiswender H., Ellison T.T., Han Z., Roon P., LeMosy E.K. (2008). Palisade is required in the *Drosophila* ovary for assembly and function of the protective vitelline membrane. Dev. Biol..

[24] Zhang Z., Stevens L.M., Stein D. (2009). Sulfation of eggshell components by Pipe defines dorsal-ventral polarity in the *Drosophila* embryo. Curr. Biol..

[25] Kendirgi F., Swevers L., Iatrou K. (2002). An ovarian follicular epithelium protein of the silkworm (*Bombyx mori*) that associates with the vitelline membrane and contributes to the structural integrity of the follicle. FEEBS Lett..

[26] Xu Y., Fu Q., Li S., He N. (2011). Silkworm egg proteins at the germ-band formation stage and a functional analysis of BmEP80 protein. Insect Biochem. Mol. Biol..

[27] Sdralia N., Swevers L., Iatrou K. (2012). BmVMP90, a large vitelline membrane protein of the domesticated silkmoth *Bombyx mori*, is an essential component of the developing ovarian follicle. Insect Biochem. Mol. Biol..

[28] Chen A., Gao P., Zhao Q., Tang S., Shen X., Zhang G., Qiu Z., Xia D., Huang Y., Xu Y. (2013). Mutation of a vitelline membrane protein, BmEP80, is responsible for the silkworm “Ming” lethal egg mutant. Gene.

[29] Chen A., Xia D., Qiu Z., Gao P., Tang S., Shen X., Zhu F., Zhao Q. (2013). Expression of a vitelline membrane protein, BmVMP23, is repressed by bmo-miR-1a-3p in silkworm, *Bombyx mori*. FEEBS Lett..

[30] Furlong M.J., Wright D.J., Dosdall L.M. (2013). Diamondback moth ecology and management: Problems, progress, and prospects. Annu. Rev. Entomol..

[31] You M., Yue Z., He W., Yang X., Yang G., Xie M., Zhan D., Baxter S.W., Vasseur L., Gurr G.M. (2013). A heterozygous moth genome provides insights into herbivory and detoxification. Nat. Genet..

[32] Peng L., Wang L., Yang Y.F., Zou M.M., He W.Y., Wang Y., Wang Q., Vasseur L., You M.S. (2017). Transcriptome profiling of the *Plutella xylostella* (Lepidoptera: Plutellidae) ovary reveals genes involved in oogenesis. Gene.

[33] Terenius O., Papanicolaou A., Garbutt J.S., Eleftherianos I., Huvenne H., Kanginakudru S., Albrechtsen M., An C., Aymeric J.L., Barthel A. (2011). RNA interference in Lepidoptera: An overview of successful and unsuccessful studies and implications for experimental design. J. Insect Physiol..

[34] Cong L., Ran F.A., Cox D., Lin S., Barretto R., Habib N., Hsu P.D., Wu X., Jiang W., Marraffini L.A. (2013). Multiplex genome engineering using CRISPR/Cas systems. Science.

[35] Knott G.J., Doudna J.A. (2018). CRISPR-Cas guides the future of genetic engineering. Science.

[36] Hammond A., Galizi R., Kyrou K., Simoni A., Siniscalchi C., Katsanos D., Gribble M., Baker D., Marois E., Russell S. (2016). A CRISPR-Cas9 gene drive system targeting female reproduction in the malaria mosquito vector *Anopheles gambiae*. Nat. Biotechnol..

[37] Reid W., O’Brochta D.A. (2016). Applications of genome editing in insects. Curr. Opin. Insect Sci..

[38] Taning C.N.T., Van Eynde B., Yu N., Ma S., Smagghe G. (2017). CRISPR/Cas9 in insects: Applications, best practices and biosafety concerns. J. Insect Physiol..

[39] Peng L., Wang Q., Zou M.M., Qin Y.D., Vasseur L., Chu L.N., Zhai Y.L., Dong S.J., Liu L.L., He W.Y. (2020). CRISPR/Cas9-mediated vitellogenin receptor knockout leads to functional deficiency in the reproductive development of *Plutella xylostella*. Front. Physiol..

[40] Zou M.M., Wang Q., Chu L.N., Vasseur L., Zhai Y.L., Qin Y.D., He W.Y., Yang G., Zhou Y.Y., Peng L. (2020). CRISPR/Cas9-induced vitellogenin knockout lead to incomplete embryonic development in *Plutella xylostella*. Insect Biochem. Mol. Biol..

[41] Griffith C.M., Lai-Fook J. (1986). Structure and formation of the chorion in the butterfly. Calpodes. Tissue Cell.

[42] Xu Y.M. (2013). Studies on Inner Eggshell Proteins and a Lethal Egg Mutant (l-em) of the Silkworm, *Bombyx mori*. Ph.D. Thesis.

[43] Zhan S., Merlin C., Boore J.L., Reppert S.M. (2011). The monarch butterfly genome yields insights into long-distance migration. Cell.

[44] Swevers L., Iatrou K. (2003). The ecdysone regulatory cascade and ovarian development in lepidopteran insects: Insights from the silkmoth paradigm. Insect Biochem. Mol. Biol..

[45] Cavaliere V., Bernardi F., Romani P., Duchi S., Gargiulo G. (2008). Building up the *Drosophila* eggshell: First of all the eggshell genes must be transcribed. Dev. Dyn..

[46] Xu Y., Zou Z., Zha X., Xiang Z., He N. (2012). A syntenic coding region for vitelline membrane proteins in four lepidopteran insects. Insect Biochem. Mol. Biol..

[47] Mineo A., Furriols M., Casanova J. (2017). Transfer of dorsoventral and terminal information from the ovary to the embryo by a common group of eggshell proteins in *Drosophila*. Genetics.

[48] Wei D., Xu H.Q., Chen D., Zhang S.Y., Li W.J., Smagghe G., Wang J.J. (2020). Genome-wide gene expression profiling of the melon fly, *Zeugodacus cucurbitae*, during thirteen life stages. Sci. Data.

[49] Huang Y., Chen Y., Zeng B., Wang Y., James A.A., Gurr G.M., Yang G., Lin X., Huang Y., You M. (2016). CRISPR/Cas9 mediated knockout of the abdominal-A homeotic gene in the global pest, diamondback moth (*Plutella xylostella*). Insect Biochem. Mol. Biol..

[50] Zhang L., Reed R.D., Sekimura T., Nijhout H.F. (2017). A practical guide to CRISPR/Cas9 genome editing in Lepidoptera. Diversity and Evolution of Butterfly wing Patterns.

[51] Chen W., Dong Y., Saqib H.S.A., Vasseur L., Zhou W., Zheng L., Lai Y., Ma X., Lin L., Xu X. (2020). Functions of duplicated glucosinolate sulfatases in the development and host adaptation of *Plutella xylostella*. Insect Biochem. Mol. Biol..

[52] Lykke-Andersen J., Bennett E.J. (2014). Protecting the proteome: Eukaryotic cotranslational quality control pathways. J. Cell Biol..

[53] Chang Y.F., Imam J.S., Wilkinson M.F. (2007). The nonsense-mediated decay RNA surveillance pathway. Annu. Rev. Biochem..

[54] Yamashita O., Irie K. (1980). Larval hatching from vitellogenin-deficient eggs developed in male hosts of the silkworm. Nature.

[55] Larkin M.A., Blackshields G., Brown N.P., Chenna R., McGettigan P.A., McWilliam H., Valentin F., Wallace I.M., Wilm A., Lopez R. (2007). Clustal W and Clustal X version 2.0. Bioinformatics.

[56] Wang W., Scali M., Vignani R., Spadafora A., Sensi E., Mazzuca S., Cresti M. (2003). Protein extraction for two-dimensional electrophoresis from olive leaf, a plant tissue containing high levels of interfering compounds. Electrophoresis.

