# Supplementary material for: Vitelline Membrane Protein 26 Mutagenesis, Using CRISPR/Cas9, Results in Egg Collapse in Plutella xylostella
Source: Int J Mol Sci. 2022 Aug 23;23(17):9538. doi: 10.3390/ijms23179538 (PMC9455775; doi:10.3390/ijms23179538)
Supplement: Supplementary file 1 [file ijms-23-09538-s001.zip › ijms-1819053-supplementary.pdf]

Supplementary Materials File

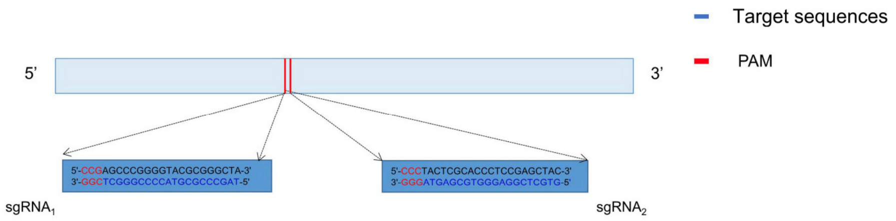


Off-target analysis

sgRNA-F1

Summary

| Target Sequence | Bulge Type | Bulge Size | Mismatch | Number of Found Targets |
|---|---|---|---|---|
| TAGCCCGCGTATCCCGGGCTNGG | X | 0 | 0 | 1 |

Details

Bulge Type | DNA bulge | RNA bulge | Mismatch | Filter | Download filtered result

| Bulge Type | Target | Chromosome | Position | Direction | Mismatches | Bulge Size |
|---|---|---|---|---|---|---|
| X | crRNA: TAGCCCGCGTATCCCGGGCTNGG<br>DNA: TAGCCCGCGTATCCCGGGCTCGG | scaffold_101 | 549314 | - | 0 | 0 |

sgRNA-F2

Summary

| Target Sequence | Bulge Type | Bulge Size | Mismatch | Number of Found Targets |
|---|---|---|---|---|
| GTAGCTCGGAGGGTGCGAGTNGG | X | 0 | 0 | 1 |

Details

Bulge Type | DNA bulge | RNA bulge | Mismatch | Filter | Download filtered result

| Bulge Type | Target | Chromosome | Position | Direction | Mismatches | Bulge Size |
|---|---|---|---|---|---|---|
| X | crRNA: GTAGCTCGGAGGGTGCGAGTNGG<br>DNA: GTAGCTCGGAGGGTGCGAGTAGG | scaffold_101 | 549342 | - | 0 | 0 |

**Figure S1.** Design and effect evaluation of sgRNA targets in the *PxVMP26* gene.

```
atg gtg ccc ctg gcg gag cca cag ttt caa ccc gca cga caa gga ccc atg caa ggc ggg
 M   V   P   L   A   E   P   Q   F   Q   P   A   R   Q   G   P   M   Q   G   G
aga cag cag cgg ttg ctc cat act acc cag cac ata ccc ccg ccg atg cag ccg ctg gaa
 R   Q   Q   R   L   L   H   T   T   Q   H   I   P   P   P   M   Q   P   L   E
gat cac aac cac ata gac aac gtg cta ccg gtg acg aca gag aag ccg cga cgt gaa gat
 D   H   N   H   I   D   N   V   L   P   V   T   T   E   K   P   R   R   E   D
aac tcc acg gtc gac gtg cac gag ctc ctc cac gct ctc ggc atc gat gag aat ggt cac
 N   S   T   V   D   V   H   E   L   L   H   A   L   G   I   D   E   N   G   H
gaa gat aag gat cac tct cga gca gct gcg agg gac tac gac tac ccg ccg agc ccg ggg
 E   D   K   D   H   S   R   A   A   A   R   D   Y   D   Y   P   P   S   P   G
tac gcg ggc tac cct ccc tac tcg cac cct ccg agc tac ggg tac cag cag cgg ccc agc
 Y   A   G   Y   P   P   Y   S   H   P   P   S   Y   G   Y   Q   Q   R   P   S
tac gcg cca aac tac ccg ccg agc tcg cct tat cct tcg tat ggt ccg cca ccg ccg tac
 Y   A   P   N   Y   P   P   S   S   P   Y   P   S   Y   G   P   P   P   P   Y
cac gag caa cca tcc tac cat cag ccg ccg ccc tcc gcc tac act ccg ccg ctg gaa gca
 H   E   Q   P   S   Y   H   Q   P   P   P   S   A   Y   T   P   P   L   E   A
cac cac agc gcg ccc tca tcc aaa cta aaa cta gtg gag atc ccg gat ctt gtc aag ccg
 H   H   S   A   P   S   S   K   L   K   L   V   E   I   P   D   L   V   K   P
ctg gcg tca aaa gtt gcc ggc aaa gtg agt gga tta atc ggc cta gtg ctc acc ctt ctc
 L   A   S   K   V   A   G   K   V   S   G   L   I   G   L   V   L   T   L   L
acc ggc tca acg ggc gac gta gaa ctc aaa ggg ttc aag gac atc gta ata aac gga ata
 T   G   S   T   G   D   V   E   L   K   G   F   K   D   I   V   I   N   G   I
gtt aag ccc ttg tta att gcc aag ggg gga tta aag agt ctg ata agc aag ttg gct ata
 V   K   P   L   L   I   A   K   G   G   L   K   S   L   I   S   K   L   A   I
cca gtg ata tcg ttg ttg ttg att aac ttg gaa gtg ctg att aca gtt tgg tgg ttg tgg
 P   V   I   S   L   L   L   I   N   L   E   V   L   I   T   V   W   W   L   W
gag gag tgt cct gag ccc gta cat gct cat gca gca tac cca gca tat cca agg cct gga
 E   E   C   P   E   P   V   H   A   H   A   A   Y   P   A   Y   P   R   P   G
tat ggc tac tga
 Y   G   Y   -
```

**Figure S2.** Amino acid sequence of the *PxVMP26* gene.
